# Effects of* Hibiscus Sabdariffa* Calyces Aqueous Extract on the Antihypertensive Potency of Captopril in the Two-Kidney-One-Clip Rat Hypertension Model

**DOI:** 10.1155/2019/9694212

**Published:** 2019-07-17

**Authors:** Shinta Ayu Nurfaradilla, Fadlina Chany Saputri, Yahdiana Harahap

**Affiliations:** ^1^Graduate Program, Faculty of Pharmacy, Universitas Indonesia, Kampus UI Depok 16424, Indonesia; ^2^Laboratory of Pharmacology, Faculty of Pharmacy, Universitas Indonesia, Kampus UI Depok 16424, Indonesia; ^3^Laboratory of Bioavailability and Bioequivalency, Faculty of Pharmacy, Universitas Indonesia, Kampus UI Depok 16424, Indonesia

## Abstract

*Hibiscus sabdariffa* aqueous extract (HS) is often used as complementary therapy for hypertension. However, some studies have shown that coadministration with a conventional antihypertensive drug can affect drug potency. We compared the effects of HS plus captopril (CAP) coadministration to HS and CAP administration alone on blood pressure and renin-angiotensin-aldosterone system (RAAS) biomarkers in the rat two-kidney-one-clip (2K1C) model of hypertension. Male Sprague Dawley rats were randomly divided into seven groups (n=6/group), a normal control (SHAM) group, and six 2K1C groups. In 2K1C animals, hypertension was induced using a stainless microclip (inner diameter of 0.20 mm). Four weeks after 2K1C surgery, blood pressure was significantly higher than in the SHAM group. Then, model rats were randomly divided into negative control (2K1C, no treatment), positive control (4.5 mg captopril/200 g body weight [BW] orally [p.o.]), HS alone (30 mg/200 g BW; p.o.), and 3 co-treatment groups receiving HS (15, 30, or 60 mg/200 g BW; p.o.) plus 4.5 mg/200 g BW captopril. The treatments were performed for two weeks. Blood pressure was significantly reduced by all the drug treatments to near the level of SHAM controls. Plasma renin level, serum angiotensin converting enzyme (ACE) activity, and plasma angiotensin II level were also significantly elevated in the 2K1C group compared to the SHAM group. Both serum ACE activity and plasma angiotensin II level were significantly reduced to near SHAM group levels by all the drug treatments.* Hibiscus sabdariffa *aqueous extract alone can reduce blood pressure. This extract appears could be used as a supplement with captopril but may not provide any additional benefit.

## 1. Introduction

Hypertension increases the risks of mortality from cardiovascular, renal, and neurological diseases and, thus, has become a major global health concern [1]. Effective management of hypertension often requires combination therapy [2, 3]. In addition to conventional antihypertensive drugs (e.g., diuretics, angiotensin converting enzyme [ACE] inhibitors, and calcium channel blockers), there are several herbal formulations with well documented antihypertensive effects, and use of these agents has increased in the last few decades among patients of all socioeconomic backgrounds [2]. However, less than 40% of patients who combine herbal preparations with conventional antihypertensive drug therapy do not inform their doctor and so may be unaware of certain deleterious side effects or herbs-drug interactions. Furthermore, there is a general lack of reliable information regarding such potential interactions [4].

*Hibiscus sabdariffa *is widely used as a herbal antihypertensive [5, 6] and frequently as complementary therapy [7, 8]. The antihypertensive efficacy of* Hibiscus sabdariffa *extract has been documented in both preclinical studies and randomized control trials [2, 5, 8, 9]. Anthocyanin and quercetin were the components in* Hibiscus sabdariffa *extract responsible for the antihypertensive effect [2, 5, 9, 10]. However, several studies have found that coadministration of* Hibiscus sabdariffa *extract with conventional drugs such as acetaminophen, chloroquine, hydrochlorothiazide, simvastatin, and ciprofloxacin produces interactions and influences the efficacy of the primary drug [2, 11–14]

There is no available information regarding the effects of* Hibiscus sabdariffa *extract coadministration with the frequently prescribed ACE inhibitor captopril on blood pressure and biomarkers of the renin-angiotensin-aldosterone system (RAAS). This study compared the antihypertensive effects of captopril alone to coadministration of* Hibiscus sabdariffa *aqueous extract plus captopril on blood pressure and RAAS biomarkers such as plasma renin level, serum ACE activity, and plasma angiotensin II level in the two-kidney-one-clip (2K1C) rat model of hypertension.

## 2. Materials and Methods

### 2.1. Preparation and Chemical Characterization of Hibiscus Sabdariffa Extract

The calyces of* Hibiscus sabdariffa *L (Malvaceae) used to produce the aqueous extract were purchased from the Research Institute for Medicinal and Aromatic Plants (Bogor, Indonesia). Samples were confirmed as* Hibiscus sabdariffa *L by the Indonesian Institute of Sciences Center for Plant Conservation Botanic Gardens (Bogor, Indonesia, reference number B-2306/IPH.3/KS/VII/2018). The aqueous extraction process was performed by the Research Institute for Medicinal and Aromatic Plants (Bogor, Indonesia). Extraction was performed by maceration at 50°C for 6–7 hours with ratio between simplicia and water 1:6. The aqueous extract then evaporated until the honey-like form. The yield of extract was 35.4%. The honey-like form extracts then were stored in 4 – 10°C. The chemical contents were determined using an Agilent 1200 Series HPLC-0053 system (Agilent Technologies, Santa Clara, CA) equipped with a diode-array detector (Agilent serial no. DE60555816) and C18 column (Inertsil ODS-3, 5.0 *μ*m, 4.6 × 150 mm; Japan). Chemical constituents were separated using a gradient mixture of 10% HCOOH in water and acetonitrile (ACN) as the mobile phase [15]. The extract contained both delphinidin 3-sambubioside (peak elution at 3.372 min) and cyanidin 3-sambubioside (peak elution at 4.337 min), which is known to act as ACE inhibitors [10]. The chromatogram of delphinidin 3-sambubioside and cyanidin 3-sambubioside will be published elsewhere. Total anthocyanin concentration was evaluated by spectrophotometry UV-Vis at 510 and 700 nm, respectively. By using this method, it was observed that the total anthocyanin contained in the extract was 0,051%.

### 2.2. Preparation of Captopril Suspension

The captopril used in this experiment is captopril 25 mg generic tablet which marketed in Indonesia. The rats will be given 4.5 mg/200 g BW of captopril suspension. In this study, every 200 g BW of rats will be given 1 mL of captopril suspension. Therefore, every 1 mL of the suspension contains 4.5 mg captopril active substance. Captopril suspension was prepared by suspending the amount of captopril powder which equivalent with the amount of captopril active substance needed in 0.5% CMC solution. The amount of captopril powder which will be suspended was calculated by weighing 20 tablets of captopril; therefore the average weight of each tablet and the ratio between the weight of each tablet vs the weight of captopril active substance contained in each tablet will be known. The tablets then crushed into powder and the calculated amount of powder will be weighed and suspended in 0.5% CMC solution. The suspension was prepared daily and stored in amber glass vial to protect it from light.

### 2.3. Experimental Animals

Normotensive male Sprague Dawley rats weighing 150–250 g were obtained from Bogor Agricultural Institute (IPB; Bogor, Indonesia). The rats were housed in a well-ventilated animal room under controlled ambient temperature (25°C ± 5°C) and regular 12 h:12 h light:dark cycle with ad libitum access to food and water. The Ethics Committee of the Faculty of Medicine, Universitas Indonesia, approved the study protocol (Jakarta, Indonesia, reference number 0643/UN2.F1.ETIK/2018).

### 2.4. Induction of Hypertension

The 2K1C hypertension model was established as follows. The rats were anesthetized by intramuscular injection of 50 mg/mL ketamine at a dose of 120 mg/kg body weight (BW). After shaving back hair and sterilizing the skin, an incision was made on the left side of the rat's body posterior. The left renal artery was identified and clipped using a stainless steel microclip (ID: 0.20 mm, Department of Mechanical Engineering Universitas Indonesia), to partially occlude renal perfusion. The incision was closed by suturing the muscle and skin with 3-0 sterile surgical PLAIN Catgut thread [16–18]. Sham-operated rats served as normal controls. This SHAM group received the same surgical procedures except for clipping of the renal artery. The rats were allowed to recover for one week. Blood pressure was measured weekly using a CODA noninvasive Tail-cuff Blood Pressure System (Kent Scientific, Torrington, CT).

### 2.5. Drug Treatments

Forty-two rats were divided randomly into seven groups (n = 6/group) as follows. Group I was the sham-operated (SHAM) group and served as a normal control. Group II consisted of untreated hypertension (2K1C) model rats and served as a negative control. Group III received oral captopril (CAP, from 25 mg generic tablets) in suspension at 4.5 mg/200 g BW as a positive control, while Group IV received* Hibiscus sabdariffa *aqueous extract (HS) at 30 mg/200 g BW (p.o.). Finally, Groups V, VI, and VII were coadministered extract at 15, 30, or 60 mg/200 g BW (p.o.) plus captopril 4.5 mg/200 g BW (p.o.) (referred to as groups CD 1, CD 2, and CD 3, respectively). The suspensions of extract and captopril were prepared daily. All the treatments were performed for two weeks. After the treatment regimens, blood pressure, plasma renin level, serum ACE activity, and plasma angiotensin II level were measured as described in detail below.

The dose of* Hibiscus sabdariffa *aqueous extract for HS and CD2 groups was chosen by reference to the daily dose of brewed HS drink used in a randomized controlled trial by Nwachukwu et al. (2015) and Herrera-Arellano et al. (2004), while doses 1 and 3 were half and two times of the daily dose, respectively.

Plasma renin was estimated using the Cusabio Rat Renin Elisa Kit (no. CSB-E08702t, Cusabio, Wuhan, China) and plasma angiotensin II using the Cusabio ANG-II Elisa Kit (no. CSB-04494r). Serum ACE activity was measured by UV-Vis spectrophotometry (Shimadzu, Kyoto, Japan) using hippuryl-his-leu (HHL) as the synthetic substrate [19]. Briefly, 100 *μ*L serum was added to 150 *μ*L of 5 mM HHL in phosphate buffered saline (pH 8.3) and the mixture incubated at 37°C for 30 min. Then, 0.25 mL of 1 N HCl was added to terminate the enzymatic reaction. The hippuric acid formed by the reaction between ACE and HHL was extracted in 1.5 mL ethyl acetate by vortexing for 30 s. Clear 1-mL aliquots from the ethyl acetate layer were transferred to another tube and heated to evaporate the solvent. The hippuric acid residue was redissolved in 1.0 mL distilled water and absorbance measured at 228 nm.

### 2.6. Statistical Analysis

All data are presented as mean ± SD. SPSS v24.0 (IBM, Armonk, NY) was used to conduct statistical analyses. Multiple group means were compared by one-way analysis of variance (ANOVA) or Kruskal−Wallis test. A P < 0.05 (two-tailed) was considered statistically significant for all tests.

## 3. Results

### 3.1. Effects of Hibiscus Sabdariffa Aqueous Extract, Captopril, and Cotreatment on Blood Pressure in 2K1C Model Rats

**Figure 1:**
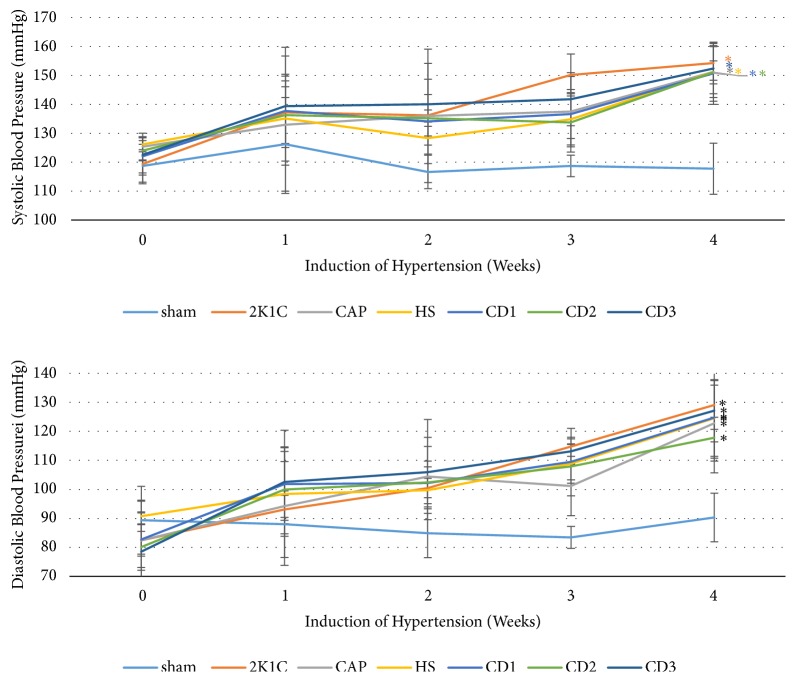
Development of hypertension following 2K1C model induction (2K1C, CAP, HS, and CD 1−CD 3 groups) compared to the SHAM surgery group. All groups except the 2K1C negative control group began receiving the specified postsurgical drug treatment (see Methods) on week 4. *∗*P < 0.05 compared to SHAM group at week 4.

Both systolic and diastolic blood pressure were rose progressively over 4 weeks after surgery in 2K1C model rats compared to the SHAM group (p < 0.05, Figure 1). There were no significant differences among model rat groups (2K1C, CAP, HS, CD 1, CD 2, and CD 3) prior to the specified postsurgical treatments.

**Figure 2:**
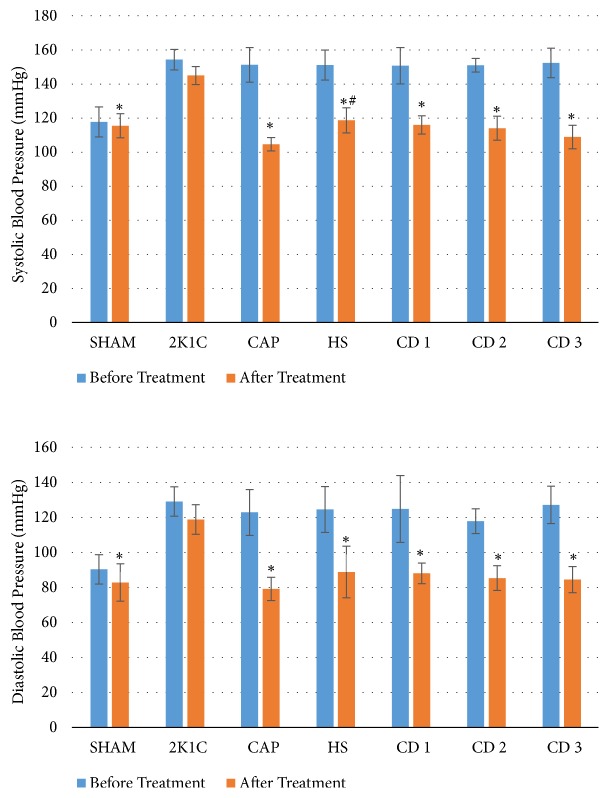
Blood pressure values before and after drug treatment (n = 6 rats/group). *∗*P < 0.05 compared to the 2K1C group; ^#^P < 0.05 compared to the CAP groups. SHAM: normal control; 2K1C: negative control (no treatment); CAP: positive control (4.5 mg/200 g BW captopril; p.o.); HS:* Hibiscus sabdariffa *extract (30 mg/200 g BW; p.o.); CD 1: coadministration of dose 1 (*Hibiscus sabdariffa *extract 15 mg/200 g BW + captopril 4.5 mg/200 g BW; p.o.); CD 2: coadministration of dose 2 (*Hibiscus sabdariffa *extract 30 mg/200 g BW + captopril 4.5 mg/200 g BW; p.o.); CD 3: coadministration of dose (*Hibiscus sabdariffa *extract 60 mg/200 g BW + captopril 4.5 mg/200 g BW; p.o.).

After two weeks, both systolic and diastolic blood pressure were significantly reduced in all the drug treatment groups (CAP, HS, CD 1, CD 2, and CD 3) compared to the untreated 2K1C group (p < 0.05, Figure 2). Systolic blood pressure was also significantly lower in the CAP group than in the HS group following treatment (p < 0.05, Figure 2), indicating that captopril alone was more efficacious for blood pressure reduction than 30 mg/200 g BW* Hibiscus sabdariffa *extract alone.

Coadministration of* Hibiscus sabdariffa *extract and captopril did not induce a further reduction in blood pressure compared to captopril alone. In fact, there were no significant differences in diastolic blood pressure among SHAM, CAP, HS, CD 1, CD 2, and CD 3 groups (p > 0.05, Figure 2). All the drug treatments reversed 2K1C-induced hypertension to near SHAM group levels.

### 3.2. Effects of Hibiscus Sabdariffa Aqueous Extract, Captopril, and Cotreatment on Plasma Renin, Serum ACE Activity, and Plasma Angiotensin II

**Figure 3:**
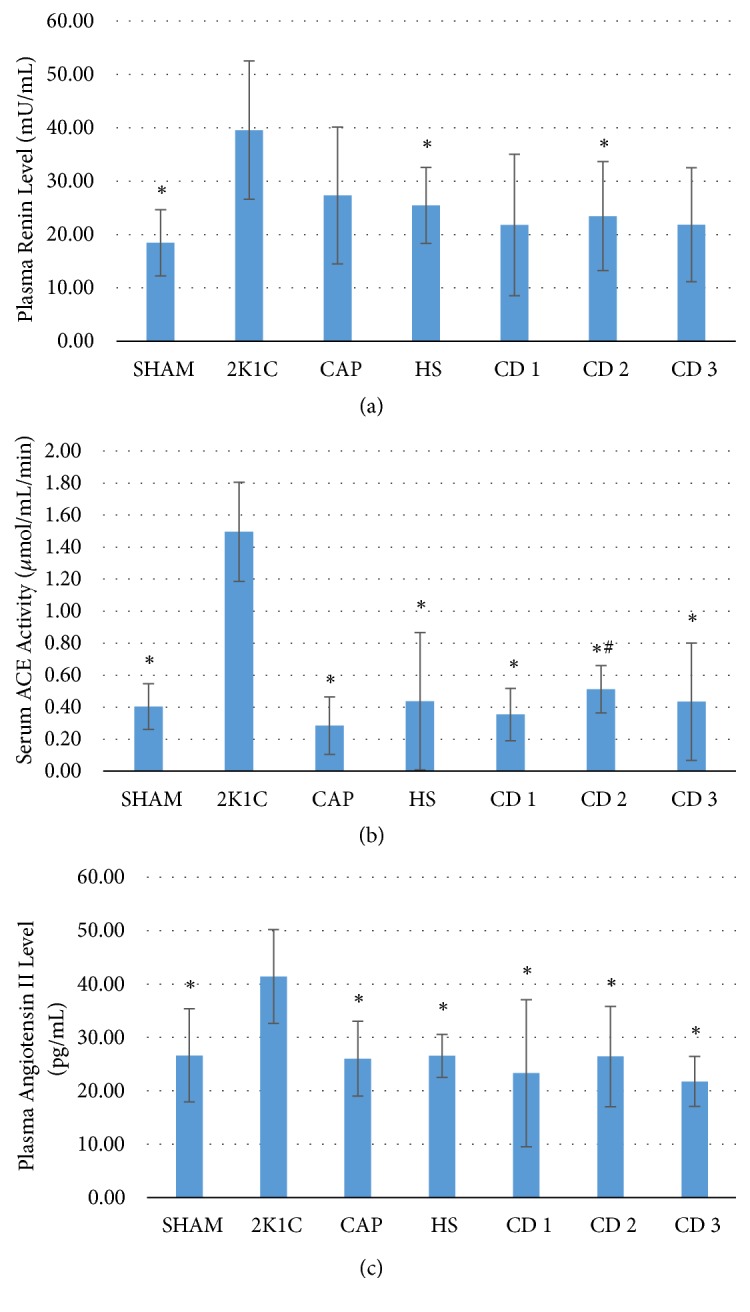
Effects of 2K1C surgery and subsequent antihypertensive drug treatments on plasma renin (a), serum ACE activity (b), and plasma angiotensin II (c). SHAM: normal control; 2K1C: negative control (no treatment); CAP: positive control (4.5 mg/200 g BW captopril; p.o.); HS:* Hibiscus sabdariffa *extract (30 mg/200 g BW; p.o.); CD 1: coadministration of dose 1 (*Hibiscus sabdariffa *extract 15 mg/200 g BW + captopril 4.5 mg/200 g BW; p.o.); CD 2: coadministration of dose 2 (*Hibiscus sabdariffa *extract 30 mg/200 g BW + captopril 4.5 mg/200 g BW; p.o.); CD 3: coadministration of dose (*Hibiscus sabdariffa *extract 60 mg/200 g BW + captopril 4.5 mg/200 g BW; p.o.). *∗*P < 0.05 compared to the 2K1C group; ^#^P < 0.05 compared to the CAP group.

Plasma renin level was significantly elevated in the 2K1C group compared to the SHAM group and significantly reduced by* Hibiscus sabdariffa *aqueous extract alone and by the CD 2 cotreatment regimen (p > 0.05, Figure 3(a)). Serum ACE activity was also significantly elevated in the 2K1C group compared to the SHAM group but was reduced by all the drug treatment regimens (CAP, HS, CD 1, CD 2, and CD 3 groups) (p < 0.05). A significant difference was also found between CAP and CD 2 group (Figure 3(b)). Finally, plasma angiotensin II was higher in the 2K1C group compared to the SHAM group and was significantly reduced by all the drug treatments (p < 0.05). There were no significant differences among the drug treatment groups or between the drug treatment groups and the SHAM group (p > 0.05, Figure 3(c)).

## 4. Discussion

*Hibiscus sabdariffa* aqueous extract is often used as complementary therapy for management of hypertension [5]; however, several studies have found interactions with conventional drug [2, 11–14]. In this study, we investigated the potential effects of* Hibiscus sabdariffa *aqueous extract coadministration on captopril by comparing blood pressure and RAAS biomarkers among captopril, HS, and cotreatment groups in the rat 2K1C hypertension model. The 2K1C model was chosen because hypertension is due mainly to upregulation of the RAAS system, so model rats respond well to ACE inhibitors such as captopril. In this model, the left renal artery is clipped by a stainless steel microclip (inner diameter: 0.20 mm) without directly damaging the kidney. The loss of perfusion induces the production of renin by the kidney. Renin in turn transforms angiotensinogen into angiotensin I, which is then converted into angiotensin II by ACE, resulting in hypertension [20, 21].

Consistent with this mode of action, captopril reduced both systolic and diastolic blood pressure reliably to near SHAM group levels. In fact, captopril alone induced the greatest reduction in blood pressure compared to* Hibiscus sabdariffa *extract alone and coadministration of the two agents, although the difference did not reach significance for most regimens. Therefore,* Hibiscus sabdariffa *extract did not appear to substantially diminish or improve the therapeutic potency of captopril, possibly because the active ingredients target the same pathway (RAAS).

In addition to ACE inhibition,* Hibiscus sabdariffa *extract also enhances cellular antioxidant capacity by increasing intracellular glutathione (GSH) [22]. However, the sulfhydryl group of GSH could bind free captopril in the circulation and dissociate the complex of captopril with albumin to form mixed disulfide captopril. This could in turn lower the antihypertensive potency of captopril.* Hibiscus sabdariffa *extract also contains quercetin. Quercetin could reduce the expression of PEPT1 transporter which has essential role in absorption of captopril. Therefore, it could lower the amount of captopril absorbed in to blood circulation [23, 24]. However, the ACE inhibitory activity of* Hibiscus sabdariffa *extract may compensate for this effect [5, 10], as evidenced by the numerical augmentation of captopril antihypertensive potency by cotreatment with increasing* Hibiscus sabdariffa *extract doses.

Plasma renin level was significantly elevated by 2K1C, likely by increasing renal production in response to loss of perfusion. All the treatments reduced plasma renin, although the decrease reached significance only with* Hibiscus sabdariffa *extract alone and one coadministration regimen (CD 2). The lack of a significant reduction in the CAP group may result from negative feedback from inhibition of angiotensin II formation, which would affect the glomerular filtration rate (GFR) and the amount of sodium delivered to the macula densa, thereby stimulating renin release [25]. All the treatments significantly reduced serum ACE activity, consistent with the known actions of captopril [26] and the active ingredients in* Hibiscus sabdariffa *extract [10]. Plasma angiotensin II level was also reduced by all the treatments, again consistent with the inhibitory actions of* Hibiscus sabdariffa *extract and captopril on ACE activity [20, 21]. All three markers were weakly correlated with blood pressure reduction by* Hibiscus sabdariffa *extract; however, possibly due to other pharmacological effects such as diuretic activity and vascular relaxation by inhibition of calcium (Ca^2+^) influx and endothelium-derived nitric oxide-cGMP relaxant pathways [2, 5, 27].

## 5. Conclusions

Coadministration of* Hibiscus sabdariffa *extract does not influence the antihypertensive potency of captopril or its effects on biomarkers of the renin-angiotensin-aldosterone system (RAAS). While* Hibiscus sabdariffa *extract does not negatively influence captopril action, it may not provide additional antihypertensive benefit.

## Data Availability

The data used to support the findings of this study are available from the corresponding author upon request.

## Supplementary Materials

Supplementary Materials Chemical profile of* H. sabdariffa* L. extracts. The chemical profile of* H. sabdariffa* L. extract was determined using an Agilent Technologies 1200 Series HPLC-0053 system equipped with a diode-array detector (serial no. DE60555816) and a C18 column (Inertsil ODS-3; 5.0 *µ*m, 4.6 × 150 mm, Japan). The compounds were separated at a flow rate of 0.5 mL/min using two gradient programs (RodriguezMedina et al. 2009). Gradient program 1 was a mixture of mobile phase A (ACN:1% HCOOH [90:10]) and mobile phase B (ACN), while gradient 2 was a mixture of mobile phase A (10% HCOOH in water) and mobile phase B (ACN). The sample injection volume was 20 *µ*L. Elution gradient 1 was used for HPLC separation and identification of polyphenols, HCA, and lactone, while gradient 2 was used to separate and identify anthocyanins in the* H. sabdariffa* L. aqueous extract. Polyphenols, HCA, and lactone were detected at 360 nm and anthocyanins at 520 nm (Rodriguez-Medina et al. 2009). The chromatograms of the chlorogenic acid (1), myricetin 3-arabinogalactoside (2), 5-O-caffeoylshikimic acid (3), and quercetin 3-rutinoside (4) are shown in Figure (A), while the chromatograms of delphinidin 3-sambubioside (1) and cyanidin 3-sambubioside (2) are shown in Figure (B).
