# Supplementary material for: Effects of Hibiscus Sabdariffa Calyces Aqueous Extract on the Antihypertensive Potency of Captopril in the Two-Kidney-One-Clip Rat Hypertension Model
Source: Evid Based Complement Alternat Med. 2019 Jul 17;2019:9694212. doi: 10.1155/2019/9694212 (PMC6662455; doi:10.1155/2019/9694212)
Supplement: Supplementary Materials — Chemical profile of H. sabdariffa L. extracts. The chemical profile of H. sabdariffa L. extract was determined using an Agilent Technologies 1200 Series HPLC-0053 system equipped with a diode-array detector (serial no. DE60555816) and a C18 column (Inertsil ODS-3; 5.0 µm, 4.6 × 150 mm, Japan). The compounds were separated at a flow rate of 0.5 mL/min using two gradient programs (RodriguezMedina et al. 2009). Gradient program 1 was a mixture of mobile phase A (ACN:1% HCOOH [90:10]) and mobile phase B (ACN), while gradient 2 was a mixture of mobile phase A (10% HCOOH in water) and mobile phase B (ACN). The sample injection volume was 20 µL. Elution gradient 1 was used for HPLC separation and identification of polyphenols, HCA, and lactone, while gradient 2 was used to separate and identify anthocyanins in the H. sabdariffa L. aqueous extract. Polyphenols, HCA, and lactone were detected at 360 nm and anthocyanins at 520 nm (Rodriguez-Medina et al. 2009). The chromatograms of the chlorogenic acid (1), myricetin 3-arabinogalactoside (2), 5-O-caffeoylshikimic acid (3), and quercetin 3-rutinoside (4) are shown in Figure (A), while the chromatograms of delphinidin 3-sambubioside (1) and cyanidin 3-sambubioside (2) are shown in Figure (B). [file 9694212.f1.docx]

## Chemical profile of H. sabdariffa L. extracts

The chemical profile of *H. sabdariffa* L. extract was determined using an Agilent Technologies 1200 Series HPLC-0053 system equipped with a diode-array detector (serial no. DE60555816) and a C18 column (Inertsil ODS-3; 5.0 µm, 4.6 × 150 mm; Japan). The compounds were separated at a flow rate of 0.5 mL/min using two gradient programs (Rodriguez-Medina et al. 2009). Gradient program 1 was a mixture of mobile phase A (ACN:1% HCOOH [90:10]) and mobile phase B (ACN) while gradient 2 was a mixture of mobile phase A (10% HCOOH in water) and mobile phase B (ACN). The sample injection volume was 20 µL.

Elution gradient 1 was used for HPLC separation and identification of polyphenols, HCA, and lactone, while gradient 2 was used to separate and identify anthocyanins in the *H. sabdariffa* L. aqueous extract. Polyphenols, HCA, and lactone were detected at 360 nm and anthocyanins at 520 nm (Rodriguez-Medina et al. 2009). The chromatograms of the chlorogenic acid (1), myricetin 3-arabinogalactoside (2), 5-O-caffeoylshikimic acid (3), and quercetin 3-rutinoside (4) are shown in Figure 1 (A), while the chromatograms of delphinidin 3-sambubioside (1) and cyanidin 3-sambubioside (2) are shown in Figure 1 (B).

**Figure 1.**

**
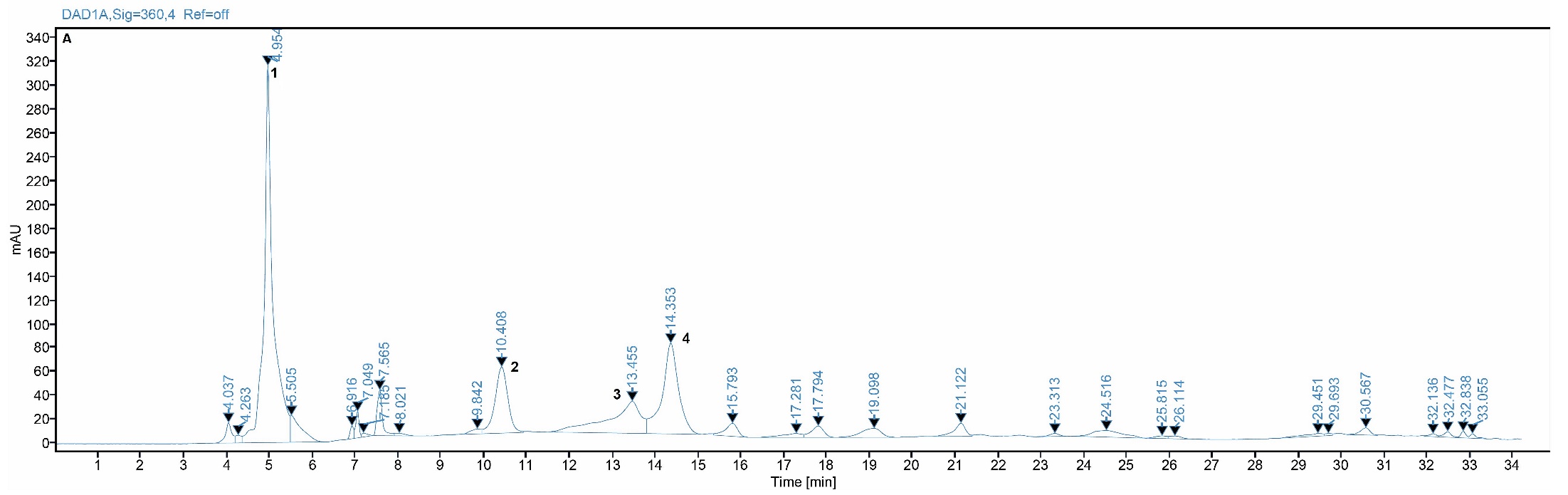
**

**
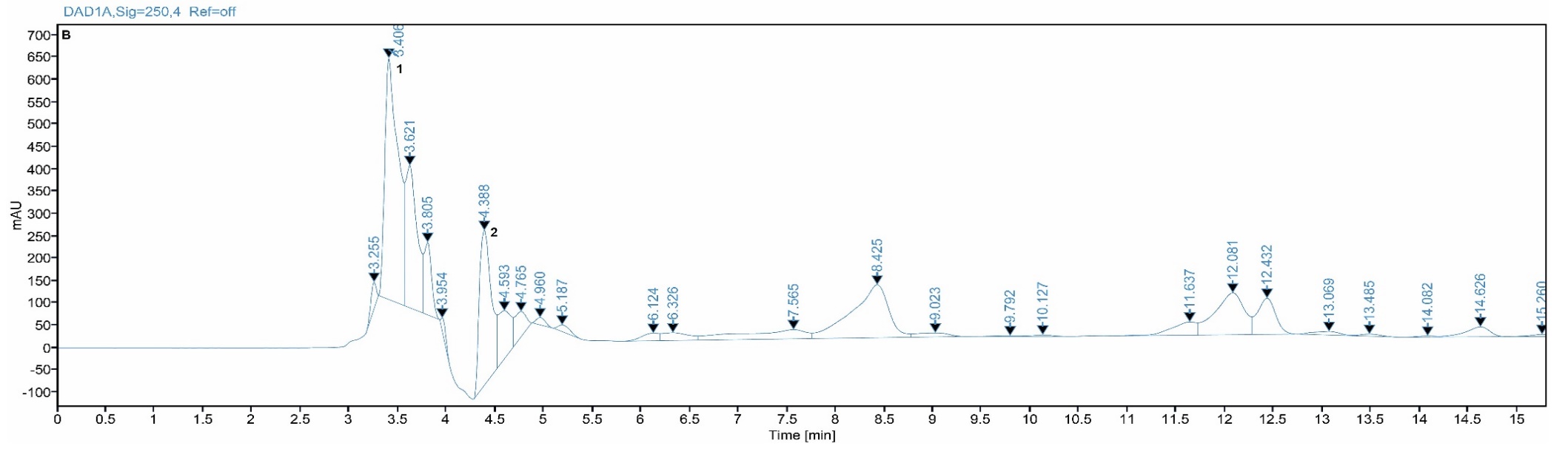
**
